# Intrinsic asymmetric ferroelectricity induced giant electroresistance in ZnO/BaTiO_3_ superlattice

**DOI:** 10.1039/d0ra09228b

**Published:** 2021-01-11

**Authors:** Ye Yuan, Yue-Wen Fang, Yi-Feng Zhao, Chun-Gang Duan

**Affiliations:** State Key Laboratory of Precision Spectroscopy, Key Laboratory of Polar Materials and Devices, Ministry of Education, Department of Electronics, East China Normal University Shanghai 200241 China; Laboratory for Materials and Structures & World Research Hub Initiative, Institute of Innovative Research, Tokyo Institute of Technology 4259 Nagatsuta, Midori Yokohama 226-8503 Japan; Collaborative Innovation Center of Extreme Optics, Shanxi University Taiyuan Shanxi 030006 China cgduan@clpm.ecnu.edu.cn

## Abstract

Here, we combine the piezoelectric wurtzite ZnO and the ferroelectric (111) BaTiO_3_ as a hexagonal closed-packed structure and report a systematic theoretical study on the ferroelectric behavior induced by the interface of ZnO/BaTiO_3_ films and the transport properties between the SrRuO_3_ electrodes. The parallel and antiparallel polarizations of ZnO and BaTiO_3_ can lead to intrinsic asymmetric ferroelectricity in the ZnO/BaTiO_3_ superlattice. Using first-principles calculations we demonstrate four different configurations for the ZnO/BaTiO_3_/ZnO superlattice with respective terminations and find one most favorable for the stable existence of asymmetric ferroelectricity in thin films with thickness less than 4 nm. Combining density functional theory calculations with non equilibrium Green's function formalism, we investigate the electron transport properties of SrRuO_3_/ZnO/BaTiO_3_/ZnO/SrRuO_3_ FTJ and SrRuO_3_/ZnO/BaTiO_3_/SrRuO_3_ FTJ, and reveal a high TER effect of 581% and 112% respectively. These findings provide an important insight into the understanding of how the interface affects the polarization in the ZnO/BaTiO_3_ superlattice and may suggest a controllable and unambiguous way to build ferroelectric and multiferroic tunnel junctions.

## Abstract 2

We combine the piezoelectric wurtzite ZnO and the ferroelectric (111) BaTiO_3_ as a hexagonal closed-packed structure and report a systematic study on the ferroelectric behavior induced by the interface and the transport properties between electrodes.

## Graphical abstract

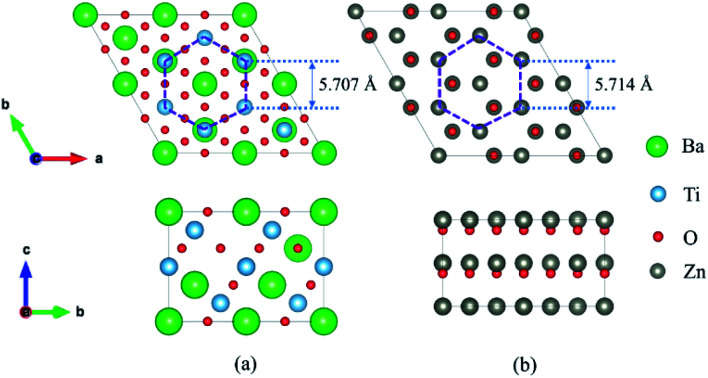

## Introduction

The past decades have witnessed an explosion in the design of ferroelectric tunnel junctions (FTJs) with the aim of accelerating their commercial applications into nonvolatile information devices.^1–13^ Switching the ferroelectric polarization gives rise to a dramatic change of the tunneling electroresistance (*i.e.*, TER effect),^10^ making it possible to nondestructively read out the polarization state that carries information. Incorporating ferroelectric and piezoelectric components could help to construct a ferroelectric tunnel junction and show a TER effect.^14–17^ Coupling between the piezoelectric ZnO and the ferroelectric BaTiO_3_ may cause bistable ferroelectric polarization orientation.^18–22^ The piezoelectric ZnO has an inherent polarization which is difficult to reverse using an electric field, but the polarization of ferroelectric BaTiO_3_ can be reversed in experiments. The parallel or antiparallel polarizations of ZnO and BaTiO_3_ may lead to intrinsic asymmetric ferroelectricity in the ZnO/BaTiO_3_ superlattice which is similar to that found in tricolor superlattices.^23^ However, there are few studies on the transport properties of ZnO/BaTiO_3_ heterostructures both in theory and experiment. In addition, BaTiO_3_ can be stacked along the [111] direction^24–26^ and can provide corrugated honeycomb interfaces which can stack closely with wurtzite ZnO. This stacking may make FTJs based on ZnO/BaTiO_3_ thinner than those currently available. The research of ZnO/BaTiO_3_ heterostructure which combined wurtzite ZnO and (111) BaTiO_3_ is relatively few.^20,21^ The theoretical research is needed to reveal its atomic scale of interfaces and transport properties.

In this paper, the piezoelectric wurtzite ZnO and the ferroelectric perovskite (111) BaTiO_3_ are closed-packed with good match of the lattice constants in all the theoretical study. We consider four configurations with respective terminations and show a systematic research on the orientation of polarization induced by the interface of ZnO/BaTiO_3_/ZnO films using first-principles calculations. After comparing the four configurations, we choose one configuration which is most favorable for the stable existence of asymmetric ferroelectricity in thin films to study its transport properties. The thickness of this ZnO/BaTiO_3_ film is less than 4 nm. Combining density functional theory calculations with non equilibrium Green's function formalism, we investigate the electron transport properties of SrRuO_3_/ZnO/BaTiO_3_/ZnO/SrRuO_3_ and SrRuO_3_/ZnO/BaTiO_3_/SrRuO_3_ with a giant TER effect of 581% and 112% respectively. Compared to the previous results,^27–31^ the TER effect of 581% for the tunnel junction combined ZnO and (111) BaTiO_3_ in this article is larger than the highest TER effect of 400%^31^ for the previous BaTiO_3_-based tunnel junctions. The junctions combined ZnO and (111) BaTiO_3_ may exhibit richer and more novel properties in the future. These findings provide an important insight into the understanding of how the interface affect the polarization in ZnO/BaTiO_3_ superlattice and may suggest a controllable and unambiguous way to build ferroelectric and multiferroic tunnel junctions.

## Method of calculation

The geometry optimizations and electronic structure calculations of all models are performed within density functional theory (DFT) calculations by using the projector augmented wave (PAW) method as implemented in the Vienna *ab initio* simulation package (VASP).^32–34^ The exchange–correlation potential is treated in the generalized gradient approximation (GGA) of Perdew, Burke, and Ernzerhof (PBE).^35^ We use the energy cutoff of 500 eV for the plane wave expansion of the PAWs. A 6 × 6 × 1 Γ centered grid for *k*-point sampling is adopted for supercells in the self-consistent calculations. The Brillouin zone integrations are calculated using the tetrahedron method with Blöchl corrections.^36^ In the structural relaxations, the atomic geometries were fully optimized until the Hellmann–Feynman forces were less than 1 meV Å^−1^.

The device properties of the FTJ are calculated by using density functional theory plus non equilibrium Green's function formalism (DFT + NEGF approach)^37,38^ as implemented in the Atomistix ToolKit-Virtual NanoLab (ATK-VNL) software package.^39^ The double-ζ plus polarization basis set is employed, and a real-space mesh cutoff energy of 80 Hartree is used to guarantee the good convergence of the device configuration. The electron temperature is set at 300 K. The 5 × 5 × 101 *k* mesh is used for the self-consistent calculations to eliminate the mismatch of Fermi level between electrodes and the central region. The 35 × 35 *k* mesh is adopted during the calculations of transmission spectra. The screening effect is considered by building screening region consist of electrode extension region and surface region.

## Results and discussion

**Fig. 1:**
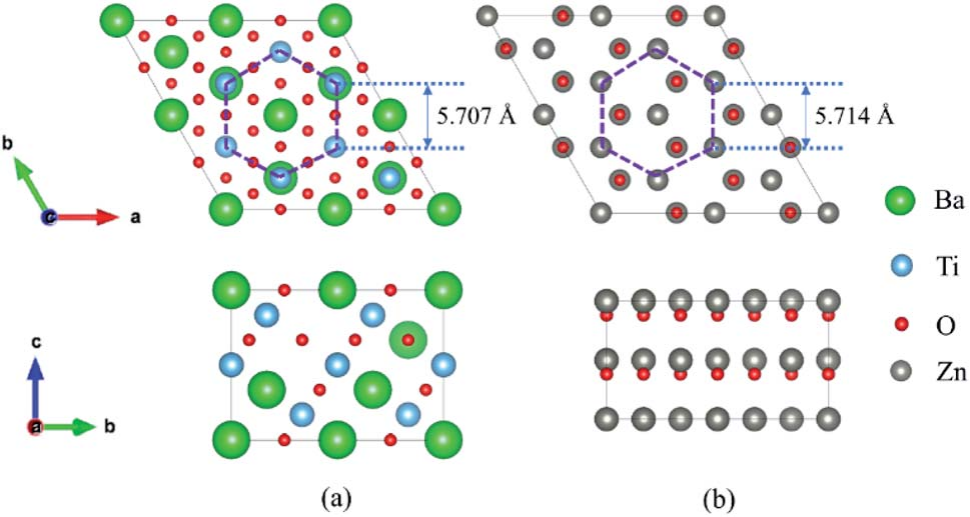
Geometry for (a) perovskite BaTiO_3_ stacking along the [111] direction and (b) a 
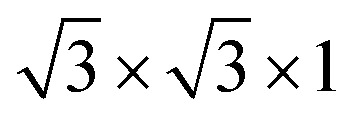
 supercell of wurtzite ZnO.

The cubic BaTiO_3_ stacking along the [111] direction has a hexagonal-like structure, where the transition metal Ti ions form the graphene-like buckled honeycomb lattice (see Fig. 1). The [111] direction in the bulk structures corresponds to the *z* direction. The theoretical lattice constant we calculated for the cubic phase of BaTiO_3_ is 4.036 Å with PBE method, and the distance between two adjacent Ti atoms in one atomic layer in the (111)-plane is 5.707 Å. The wurtzite ZnO has a hexagonal structure with theoretical in-plane lattice constant of 3.299 Å, and the distance between two adjacent Zn atoms of the honeycomb in 
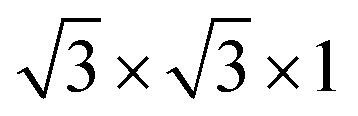
 superlattice is 5.714 Å. Hence, the (111) BaTiO_3_ and wurtzite ZnO have a very good match of the lattice constants (a mismatch is only about 0.1%) that allows layer-by-layer epitaxial growth of ZnO/BaTiO_3_ (111) multilayers with no misfit dislocation.

**Fig. 2:**
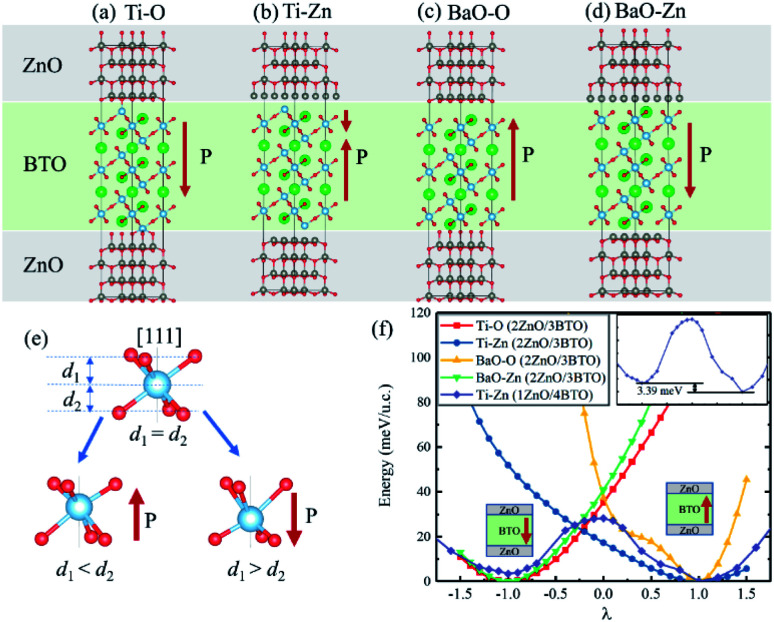
Four different configurations of the ZnO/BaTiO_3_/ZnO heterostructure with the respective terminations of (a) Ti–O, (b) Ti–Zn, (c) BaO–O and (d) BaO–Zn. (e) The ferroelectric distortion of octahedron in (111) BaTiO_3_. (f) The single well potential for ZnO/BaTiO_3_/ZnO superlattics with four different terminations of 2ZnO/3BTO and double well potential for 1ZnO/4BTO. The asymmetry is shown in the embedded figure.

We calculated ZnO/BaTiO_3_/ZnO supercell composed of two layers of ZnO and three layers of BaTiO_3_ (BTO). Four different configurations of the ZnO/BaTiO_3_/ZnO heterostructure were considered, with the respective terminations of Ti–O (Fig. 2(a)), Ti–Zn (Fig. 2(b)), BaO–O (Fig. 2(c)) and BaO–Zn (Fig. 2(d)). The direction of polarization of BaTiO_3_ was adjusted to the [111] direction under the influence of the interface and can be obtained by analyzing the relative Ti–O displacements along the [111] direction, see Fig. 2(e). The three O atoms at the top of the octahedron are on the same plane, and their vertical distance from Ti atom is *d*_1_. Similarly, *d*_2_ is the vertical distance from three O atoms at the bottom of the octahedron to Ti atom. Therefore, the ferroelectric polarization is up when *d*_1_ < *d*_2_, and is down when *d*_1_ > *d*_2_. Using this way, the polarization *P* in the four different configurations were determined, as indicated by the red arrows in Fig. 2(a–d). As shown in Fig. 2(a), the first kind of terminations of the interfaces is Ti–O, means the atomic layer of Ti in (111) BaTiO_3_ contact with O layer in ZnO. This configuration make BaTiO_3_ has a polarization pointing from top to bottom, and the polarization is very robust. The single well potential in Fig. 2(f) denotes the polarization is almost impossible to reverse. The second configuration is Ti–Zn, means the Ti layer in (111) BaTiO_3_ contact with Zn layer in ZnO. This kind of configuration make the interior polar displacements of BaTiO_3_ near the top interface have opposite orientation with that near the bottom interface, and the net polarization is pointing up. The naming rules for the other two configurations are the same as above. When we increase the thickness of BaTiO_3_ and decrease the thickness of ZnO for the Ti–Zn configuration, a double-well profile is observed in Fig. 2(f) which is a general feature of ferroelectric materials. From these results, we concluded that the Ti–Zn configuration may implement reversible FTJ relatively easier in thicker BaTiO_3_.

**Fig. 3:**
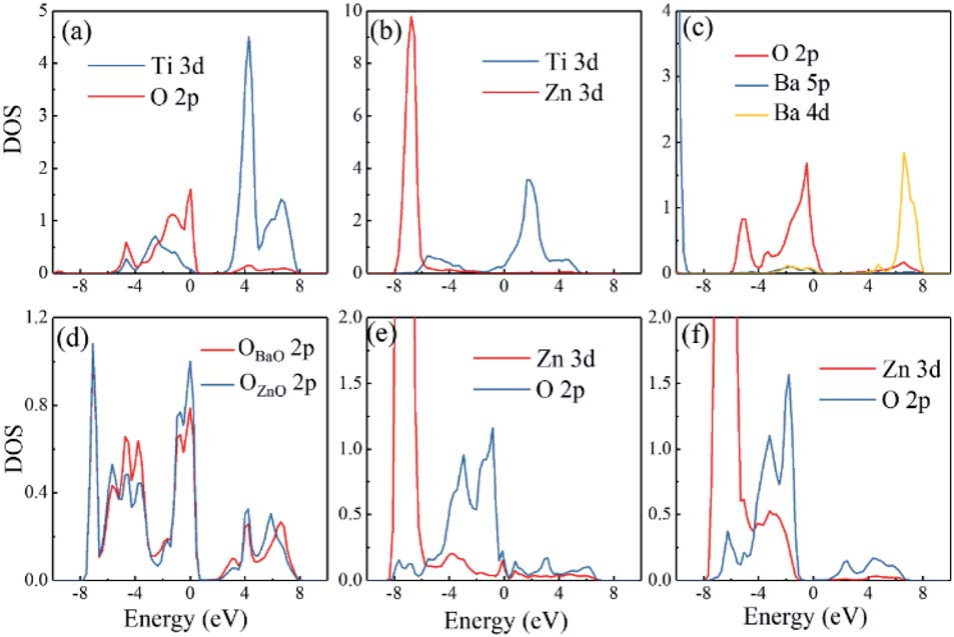
The orbital projected density of states of atoms at the interfaces for different configurations of the ZnO/BaTiO_3_/ZnO heterostructures. (a) Ti of BaTiO_3_ and O of ZnO in bottom interface of Ti–O configuration. (b) Ti of BaTiO_3_ and Zn of ZnO in bottom interface of Ti–Zn configuration. (c) Ba of BaTiO_3_ and O of ZnO in top interface of Ba–O configuration. (d) O of BaTiO_3_ and O of ZnO in bottom interface of Ba–O configuration. (e) O of BaTiO_3_ and Zn of ZnO in top interface of BaO–Zn configuration. (f) O of BaTiO_3_ and Zn of ZnO in bottom interface of BaO–Zn configuration.

**Fig. 4:**
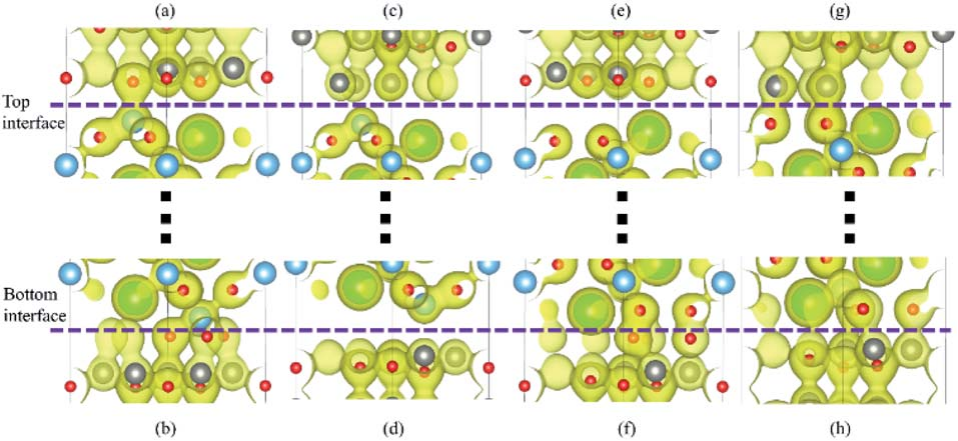
(a) The charge density of the top interfaces and bottom interfaces in four configurations. (a) Top interface for Ti–O configuration. (b) Bottom interface for Ti–O configuration. (c) Top interface for Ti–Zn configuration. (d) Bottom interface for Ti–Zn configuration. (e) Top interface for BaO–O configuration. (f) Bottom interface for BaO–O configuration. (g) Top interface for BaO–Zn configuration. (h) Bottom interface for BaO–Zn configuration.

Different interfaces between BaTiO_3_ and ZnO can induce to different polarizations of BaTiO_3_ near the interfaces. The reason for it in Ti–O configuration lies in the different vertical distance between Ti and O atoms at top and bottom interfaces. The Ti atoms from BaTiO_3_ and O atoms from ZnO are bonding in the interfaces as shown in Fig. 3(a). In the top interface, the O atoms is directly above the nearest neighbour Ti atoms, and the bond length is about 1.93 Å. However, in the bottom interface, the O atoms lies diagonally below the Ti atoms, so the vertical distance between Ti and O atoms is small although the bond length is 1.85 Å. The difference of Ti–O bonding between top interface and bottom interface can be clarified in Fig. 4(a) and (b). This asymmetry in top and bottom interfaces causes the center of positive charge to move downward. That is why the polarization is from the top interface to the bottom interface in Ti–O configuration. In Ti–Zn configuration, the Ti atoms and Zn atoms are both positive ions, the orbital projected density of states show that the overlapping between these two transition metal ions are very few. The Coulomb repulsion play a dominant role here. That is why the BaTiO_3_ near top interface and bottom interface have different polarizations. Fig. 3(c) and (d) show the situation of both top interface and bottom interface in BaO–O configuration. The results denote that the Ba atoms from BaTiO_3_ and O atoms from ZnO in top interface cannot bond because there is no overlapping between their density of states. However, the O atoms from BaTiO_3_ and O atoms from ZnO can form covalent bonds as shown in Fig. 3(d). Therefore, the center of negative charge moves downward, results in the polarization of BaTiO_3_ in BaO–O configuration from bottom interface to top interface. In the BaO–Zn configuration, the O atoms from BaTiO_3_ and Zn atoms from ZnO in both top interface and bottom interface are bonding, but the bond in top interface is stronger. As shown in Fig. 3(e) and (f), the Zn 3d orbital and O 2p orbital in top interface have overlapping from −6 eV to 0 eV, and the overlapping in bottom interface is only from −4 eV to −1 eV. Therefore, the polarization of BaTiO_3_ for BaO–Zn configuration is from top interface to bottom interface.

The more intuitive results are shown in Fig. 4(a)–(h). All the graphical charge densities have the same isosurface. It is clear that the Ti atoms from BaTiO_3_ and O atoms from ZnO are bonding in both top and bottom interfaces for Ti–O configuration, but the vertical lengths in bottom interface are shorter. The Ti atoms and Zn atoms in Ti–Zn configuration cannot bond and so do the Ba atoms and O atoms in BaO–O configuration. The bonds of O atoms and Zn atoms in top interface are stronger than that in bottom interface. The results of these charge densities are consistent with the above analysis based on the density of states.

**Fig. 5:**
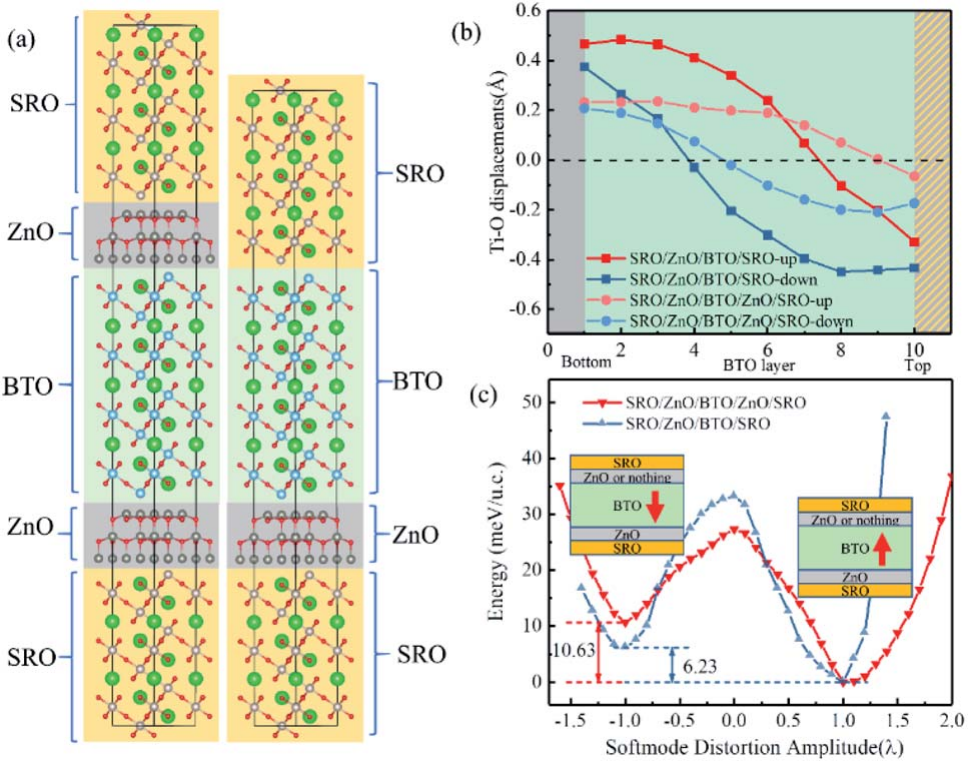
(a) The atomic structures of SrRuO_3_/ZnO/BaTiO_3_/ZnO/SrRuO_3_ and SrRuO_3_/ZnO/BaTiO_3_/SrRuO_3_ supercells. (b) Rumpling (the displacement of the Ti atoms with respect to the central of the octahedron) profiles for SrRuO_3_/ZnO/BaTiO_3_/ZnO/SrRuO_3_ and SrRuO_3_/ZnO/BaTiO_3_/SrRuO_3_ supercell. (c) Double-well potential for the supercells (we set the total energy of the two up states to 0 meV just for the convenience of discussions, but this does not mean that the energy of two up states are equal).

In Fig. 5(a), three layers of SrRuO_3_ (SRO) were interfaced to the ZnO/BTO/ZnO overlayers to form the SRO/ZnO/BTO/ZnO/SRO and SRO/ZnO/BTO/SRO heterostructures. We define the state which has polarization from bottom interface to top interface as the ‘up’ state and the opposite direction as the ‘down’ state. From the rumpling of the ‘up’ and ‘down’ states in junction structures as shown in Fig. 5(b), we can see that the ferroelectric displacements of both supercells are significantly asymmetric. For the average polarization displacements, the difference between ‘up’ and ‘down’ states are dramatic. Our calculations have found two inequivalent energy minima in each system, as clearly shown in Fig. 5(c). This is the signature of asymmetric ferroelectricity. Note that here the energy profiles are obtained by simulating the soft mode distortion of the BaTiO_3_ layer (characterized by the parameter *λ*), where we choose states with *λ* = +1 to be the lowest energy states (‘up’ states) and states with *λ* = −1 to be the metastable energy states (‘down’ states) for both two supercells. The coordinates of other λ states are linear interpolations (|*λ*| < 1) or extrapolations (|*λ*| > 1) between the coordinates of ‘up’ and ‘down’ states according to their values. Here, the thickness of ZnO/BaTiO_3_ superlattice is less than 4 nm, which is thinner than those currently available in experiment.^18–22^

**Fig. 6:**
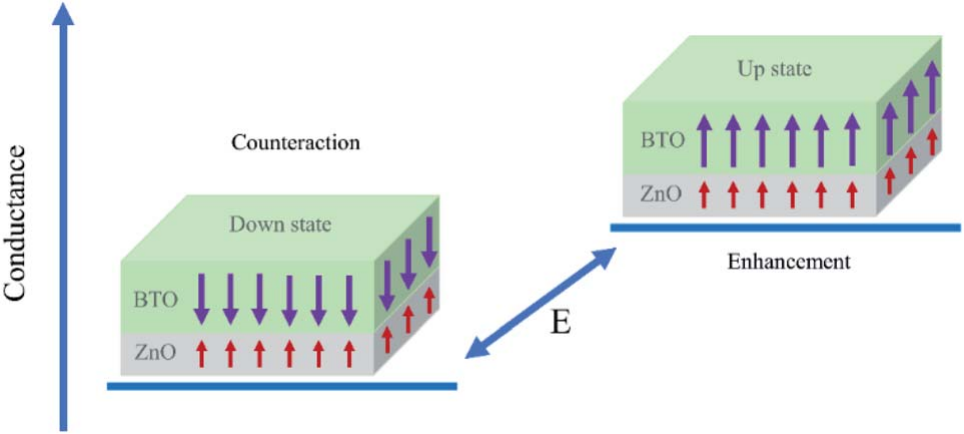
Conductance of the SrRuO_3_/ZnO/BaTiO_3_/SrRuO_3_ FTJ. The two conductance states are distinguished by polarization of BaTiO_3_.

Essentially speaking, the asymmetric ferroelectricity of ZnO/BaTiO_3_ superlattice comes from the relative orientations of polarizations of ZnO and BaTiO_3_ in supercells. When the orientations of the polarization of ZnO and BaTiO_3_ are opposite, the counteraction of the intensity of total polarization is appeared. This will result in a low conductance. The high conductance comes from the enhancement of total polarization resulting from the same orientations of the polarization of ZnO and BaTiO_3_. Fig. 6 shows schematically the two conductance levels indicating the possibility of switching between them by electric (*E*) fields.

To evaluate the performance of SRO/ZnO/BTO/ZnO/SRO and SRO/ZnO/BTO/SRO FTJs, density functional theory plus non equilibrium Green's function formalism is used to study the electrical conductance and TER effect. In our calculations, the transmission coefficients and reflection matrices are determined by matching the wave functions of the scattering region with linear combinations of propagating Bloch states in the electrodes. Because the electronic states at the EF dominate the transport properties, the zero-bias electrical conductance within the Landauer−Büttiker formula can be evaluated as1
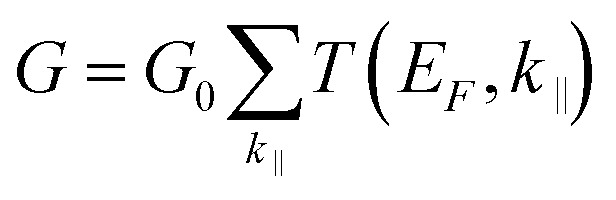
where *G*_0_ = 2 × 10^2^/ℏ is the conductance quantum, *e* is the electron charge, ℏ is the Planck's constant, and *T*(*E*_F_,*k*_‖_) is the transmission coefficient at the Fermi energy for a given Bloch wave vector *k*_‖_ = (*k*_*x*_,*k*_*y*_) in the 2D Brillouin zone. By integrating the transmission probability for states at the Fermi energy over the 2D Brillouin zone, we can calculate the total conductance (*G*). In the up state of SRO/ZnO/BTO/ZnO/SRO FTJ, *G*_up_ = 1.122 × 10^−7^*S*; by contrast, in the down state, *G*_dn_ = 1.647 × 10^−8^*S*. For SRO/ZnO/BTO/SRO FTJ, *G*_up_ = 1.771 × 10^−7^*S*, *G*_dn_ = 8.336 × 10^−8^*S*. Following the conventional definition in previous study,^40^ the TER ratio in our study is defined as2
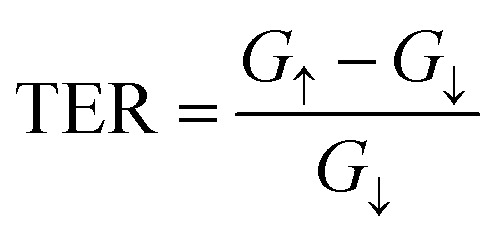


As a result, the reversal of ferroelectric polarization in the SRO/ZnO/BTO/ZnO/SRO FTJ leads to a significantly enhanced TER effect at zero bias, which is approximately about 581%. The TER effect for SRO/ZnO/BTO/SRO FTJ is 112%, which is also tremendous.

**Fig. 7:**
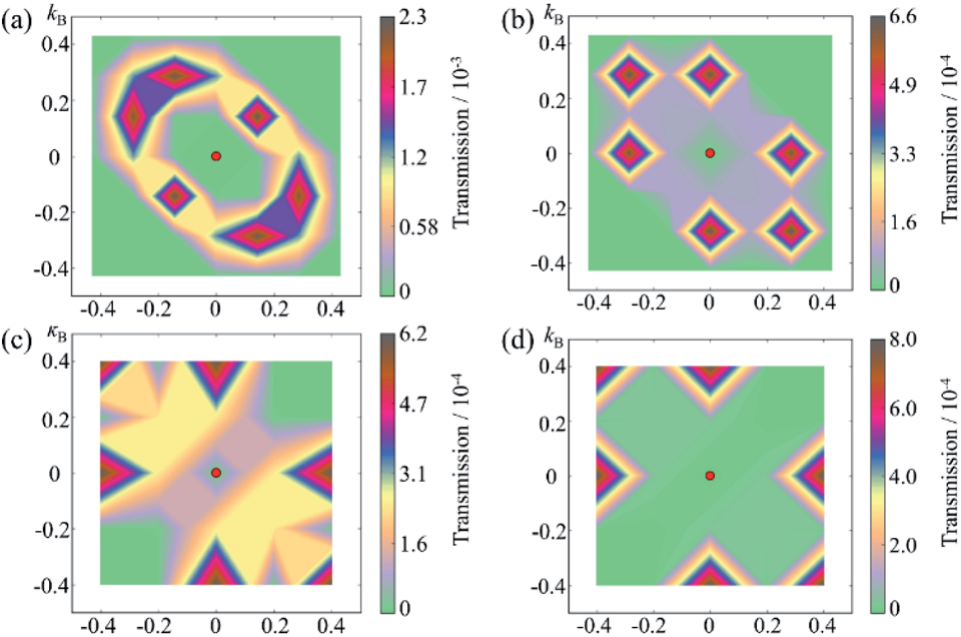
Supercell *k*_‖_-resolved transmissions in 2D Brillouin zone at the Fermi energy through the SRO/ZnO/BTO/ZnO/SRO FTJ for (a) up and (b) down states and SRO/ZnO/BTO/SRO FTJ for (c) up and (d) down states.

To understand the change in the conductance ratio during the polarization reversal, the *k*_‖_-resolved transmissions at *E*_F_ are shown in Fig. 7. In the up state of SRO/ZnO/BTO/ZnO/SRO FTJ, the transmission coming from the rhombus regions of the 2D Brillouin zone are largest, indicating the feature of resonant tunneling. We find the transmission eigenstates around this region show much smaller decay rate than those around Γ point, which is responsible for the significant transmission. The reason is that the amplitude of transmission eigenstates for this region is much larger than that for Γ point, which indicates greater transmission probability. Compared to the up state, the transmission in the down state is largely reduced, leading to lower conductance than the up state. This explains the observed giant TER effect in the SRO/ZnO/BTO/ZnO/SRO and SRO/ZnO/BTO/SRO FTJ.

## Conclusions

In conclusion, the four stacking configurations of ZnO/BTO/ZnO supercell with two layers of ZnO and three layers of (111) BTO have been studied using DFT calculations. The mechanism of polarization direction in (111) BTO influenced by the interfaces are investigated. Combining density functional theory calculations with non equilibrium Green's function formalism, two giant TER effects of 581% and 112% are observed in our newly designed SRO/ZnO/BTO/ZnO/SRO and SRO/ZnO/BTO/SRO FTJ heterostructure. The thickness of ZnO/BTO in this heterostructure is less than 4 nm. The proposed strategy in our study is applicable to design higher performance FTJs. We hope this work will stimulate the experimental endeavors of fabricating FTJs with a giant TER effect to accelerate their commercial applications into ultralow-power, high-speed, and nonvolatile nanoscale memory devices.

## Conflicts of interest

There are no conflicts to declare.

## Supplementary Material

## References

[cit1] Huang W., Fang Y. W., Yin Y., Tian B., Zhao W., Hou C., Ma C., Li Q., Tsymbal E. Y., Duan C. G., Li X. (2018). ACS Appl. Mater. Interfaces.

[cit2] Boyn S., Grollier J., Lecerf G., Xu B., Locatelli N., Fusil S., Girod S., Carretero C., Garcia K., Xavier S., Tomas J., Bellaiche L., Bibes M., Barthelemy A., Saighi S., Garcia V. (2017). Nat. Commun..

[cit3] Martin L. W., Rappe A. M. (2017). Nat. Rev. Mater..

[cit4] Jin Hu W., Wang Z., Yu W., Wu T. (2016). Nat. Commun..

[cit5] Shen X. W., Fang Y. W., Tian B. B., Duan C. G. (2019). ACS Appl. Electron. Mater..

[cit6] Lu C., Hu W., Tian Y., Wu T. (2015). Appl. Phys. Rev..

[cit7] Chanthbouala A., Garcia V., Cherifi R. O., Bouzehouane K., Fusil S., Moya X., Xavier S., Yamada H., Deranlot C., Mathur N. D., Bibes M., Barthelemy A., Grollier J. (2012). Nat. Mater..

[cit8] Garcia V., Bibes M., Bocher L., Valencia S., Kronast F., Crassous A., Moya X., Enouz-Vedrenne S., Gloter A., Imhoff D., Deranlot C., Mathur N. D., Fusil S., Bouzehouane K., Barthelemy A. (2010). Science.

[cit9] Garcia V., Fusil S., Bouzehouane K., Enouz-Vedrenne S., Mathur N. D., Barthelemy A., Bibes M. (2009). Nature.

[cit10] Zhuravlev M. Y., Sabirianov R. F., Jaswal S. S., Tsymbal E. Y. (2005). Phys. Rev. Lett..

[cit11] Tsymbal E. Y., Kohlstedt H. (2006). Science.

[cit12] Velev J. P., Duan C. G., Belashchenko K. D., Jaswal S. S., Tsymbal E. Y. (2007). Phys. Rev. Lett..

[cit13] Scott J. F. (2007). Nat. Mater..

[cit14] Yuan S., Wang J., Zhong X., Wang F., Li B., Zhou Y. (2013). J. Mater. Chem. C.

[cit15] Kohlstedt H., Pertsev N. A., Rodríguez Contreras J., Waser R. (2005). Phys. Rev. B: Condens. Matter Mater. Phys..

[cit16] Lu X., Cao W., Jiang W., Li H. (2012). J. Appl. Phys..

[cit17] Zheng Y., Woo C. H. (2009). Nanotechnology.

[cit18] Jia C., Yin X., Yang G., Wu Y., Li J., Chen Y., Zhang W. (2017). Appl. Phys. Lett..

[cit19] Aepuru R., Kankash S., Panda H. S. (2016). RSC Adv..

[cit20] Voora V. M., Hofmann T., Brandt M., Lorenz M., Ashkenov N., Grundmann M., Schubert M. (2009). Appl. Phys. Lett..

[cit21] Voora V. M., Hofmann T., Schubert M., Brandt M., Lorenz M., Grundmann M., Ashkenov N., Schubert M. (2009). Appl. Phys. Lett..

[cit22] Sekhar K. C., Silva J. P. B., Kamakshi K., Pereira M., Gomes M. J. M. (2013). Appl. Phys. Lett..

[cit23] Gao Y. C., Duan C. G., Tang X. D., Hu Z. G., Yang P., Zhu Z., Chu J. (2013). J. Phys.: Condens. Matter.

[cit24] Watanabe T., Shimada M., Aiba T., Yabuta H., Miura K., Oka K., Azuma M., Wada S., Kumada N. (2011). Jpn. J. Appl. Phys..

[cit25] Raeliarijaona A., Fu H. (2014). J. Appl. Phys..

[cit26] Eibl O., Pongratz P., Skalicky P., Schmelz H. (1987). J. Am. Ceram. Soc..

[cit27] Qian M. D., Fina I., Sanchez F., Fontcuberta J. (2019). Adv. Electron. Mater..

[cit28] Qian M., Fina I., Sánchez F., Fontcuberta J. (2019). Small.

[cit29] Abuwasib M., Lee H., Lee J.-w., Eom C.-B., Gruverman A., Singisetti U. (2019). IEEE Trans. Electron Devices.

[cit30] Andreeva N. V., Petrov A. A., Petraru A., Petukhov A. E., Rybkin A. G. (2018). Mater. Res. Express.

[cit31] Li C., Huang L., Li T., Lü W., Qiu X., Huang Z., Liu Z., Zeng S., Guo R., Zhao Y., Zeng K., Coey M., Chen J., Ariando A., Venkatesan T. (2015). Nano Lett..

[cit32] Kresse G., Furthmuller J. (1996). Comput. Mater. Sci..

[cit33] Kresse G., Furthmuller J. (1996). Phys. Rev. B: Condens. Matter Mater. Phys..

[cit34] Kresse G., Joubert D. (1999). Phys. Rev. B: Condens. Matter Mater. Phys..

[cit35] Perdew J. P., Burke K., Ernzerhof M. (1996). Phys. Rev. Lett..

[cit36] Blochl P. E., Jepsen O., Andersen O. K. (1994). Phys. Rev. B: Condens. Matter Mater. Phys..

[cit37] Taylor J., Guo H., Wang J. (2001). Phys. Rev. B: Condens. Matter Mater. Phys..

[cit38] Brandbyge M., Mozos J.-L., Ordejón P., Taylor J., Stokbro K. (2002). Phys. Rev. B: Condens. Matter Mater. Phys..

[cit39] Atomistix ToolKit version 2014.3-Virtual NanoLab version 2017.2, QuantumWise A/S, https://www.quantumwise.com

[cit40] Velev J. P., Duan C. G., Burton J. D., Smogunov A., Niranjan M. K., Tosatti E., Jaswal S. S., Tsymbal E. Y. (2009). Nano Lett..

